# Analysis of the Effects of Thermal Environment on Optical Systems for Navigation Guidance and Control in Supersonic Aircraft Based on Empirical Equations

**DOI:** 10.3390/s16101717

**Published:** 2016-10-17

**Authors:** Xuemin Cheng, Yikang Yang, Qun Hao

**Affiliations:** 1 Graduate School at Shenzhen, Tsinghua University, Shenzhen 518055, China; cheng-xm@mail.tsinghua.edu.cn (X.C.); yangyk13@mails.tsinghua.edu.cn (Y.Y.); 2 Beijing Key Lab for Precision Optoelectronic Measurement Instrument and Technology, School of Optoelectronics, Beijing Institute of Technology, Beijing 100081, China

**Keywords:** thermal analysis, finite element analysis, empirical equation, optical systems for navigation guidance and control in supersonic aircraft, computation efficiency

## Abstract

The thermal environment is an important factor in the design of optical systems. This study investigated the thermal analysis technology of optical systems for navigation guidance and control in supersonic aircraft by developing empirical equations for the front temperature gradient and rear thermal diffusion distance, and for basic factors such as flying parameters and the structure of the optical system. Finite element analysis (FEA) was used to study the relationship between flying and front dome parameters and the system temperature field. Systematic deduction was then conducted based on the effects of the temperature field on the physical geometry and ray tracing performance of the front dome and rear optical lenses, by deriving the relational expressions between the system temperature field and the spot size and positioning precision of the rear optical lens. The optical systems used for navigation guidance and control in supersonic aircraft when the flight speed is in the range of 1–5 Ma were analysed using the derived equations. Using this new method it was possible to control the precision within 10% when considering the light spot received by the four-quadrant detector, and computation time was reduced compared with the traditional method of separately analysing the temperature field of the front dome and rear optical lens using FEA. Thus, the method can effectively increase the efficiency of parameter analysis and computation in an airborne optical system, facilitating the systematic, effective and integrated thermal analysis of airborne optical systems for navigation guidance and control.

## 1. Introduction

Recent developments in aircraft technology have focused attention on the airborne optical systems of supersonic aircraft [1,2]. The temperature fields of supersonic aircraft in flight are extremely affected by the complexities of the thermal environment, such as environmental temperature, air flow, the aerodynamic heating effect and solar radiation, resulting in internal temperature gradients of the optical system. Both the geometry and performance of airborne optical components are affected [3]. For example, changes in geometry and the index of refraction can lead to failure of the optical system [4]. The thermal environment of optical systems, particularly those for navigation guidance and control that require high performance in terms of light spot size and target positioning, must therefore be taken into consideration during design.

Airborne optical systems for navigation guidance and control are comprised of two parts; the front dome and the rear internal optical lens. Xiao et al. studied the influences of a non-uniform aerodynamic flow field and aerodynamic heating on the index of refraction and profile of the dome, and assessed imaging quality degradation in the airborne optical system using a fourth-order Runge-Kutta algorithm [1]. Wang et al. presented the use of uniform and hexahedral grids generated from computational fluid dynamics for the analysis of aero-optical performance [5]. Few thermal analyses have been conducted on airborne rear optical lenses with external load changes. Based on the thermal sensitivity method, Blaurock et al. investigated optical sensitivity to temperature using finite element analysis (FEA) [6]. Applewhite et al. studied the effects of thermal gradients on the Mars Observer Camera primary mirror [4]. Most studies have focused on component thermal analyses of the front dome and the rear internal optical lens in independent temperature fields.

However, there are certain problems with this approach. (1) During flight, the dome comes into direct contact with the exterior thermal environment. The rear optical lens receives heat through conduction. Hence, a performance analysis of solely the dome or the rear optical lens will be incomplete. The airborne optical system for navigation guidance and control should be considered as a whole in any evaluation of the influence of the thermal environment on its performance; (2) The purpose of thermal analysis of optical systems is to facilitate athermal design, so an understanding of the effects of changes in flying and structural parameters on the temperature field is required; (3) During system design, integrated optical-structural-thermal analysis is repeatedly carried out for verification. A massive amount of iterative computation is required, as the analysis relies on the finite element method (FEM), the finite volume method and other numeric simulation methods when calculating the crucial system parameters. A systematic and integrated thermal analysis technology that can increase the computation efficiency of integrated optical-structural-thermal analysis is therefore required.

To achieve a systematic integrated thermal analysis of navigation guidance and control airborne optical systems, systematic deduction is proposed in this study. First, we derive relational expression between the front temperature field and the emergent ray angles, and the amplification of the results of the rear lens by accounting for the effects of the temperature field on the physical geometry and ray tracing performance of the front dome and rear optical components. The relational expressions between the front temperature field, the light spot size and the positioning precision of the rear optical lens are then found. To investigate the effects of flying parameters and system structural parameters on the temperature field, an empirical equation was then developed to describe the relationship between the front temperature gradient or rear thermal diffusion distance and basic factors such as the optical-structural nature of the airborne optical system, flight speed and flight time. Finally, using a specific system layout as an example, the factors linked with the front temperature field and the internal thermal diffusion speed were analysed. These served as the basic criteria for the performance of the rear optical system.

In summary, by using empirical equations, the system temperature field and the influence of the front temperature gradient on system performance were calculated directly, which enabled rapid integrated optical-structural-thermal analysis for achieving the crucial system parameters. Finally, experimental FEA validation indicated that this method can dramatically reduce the required analysis time.

## 2. Materials and Methods

**Figure 1:**
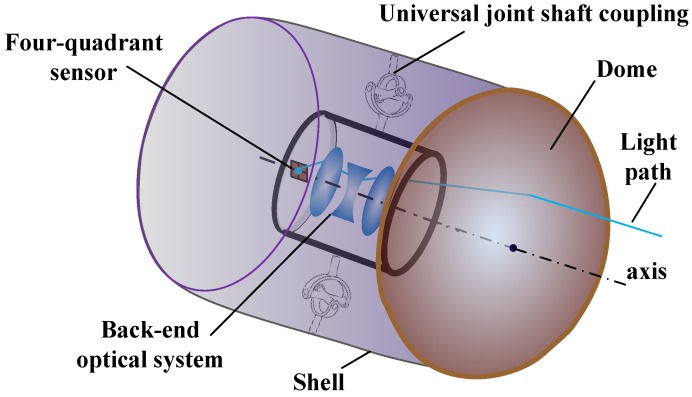
Airborne guided optical layout.

The airborne optical system for aircraft positioning, or for navigation guidance and control, consists of two parts: the front dome and the rear optical lens. Figure 1 is a schematic view of the structure. At the front is a rotational symmetric dome for light collection, and at the back is a cylindrical rotational symmetric shell filled with air. The rear optical lens is placed in the middle part of the shell and connected to it with a universal joint shaft coupling, to facilitate posture adjustment and the corresponding target detection. The main source of heat load is assumed to be aerodynamic heat, as described in Appendix A. The system is also assumed to work with an attach angle of 0, under the condition of the oncoming flow moving parallel to the axis of the optical system, as the system posture is adjusted when flying by using the positioning detection signal. Other conditions (for example, with a variable attach angle) of the flow around the modelled sensor will be the subject of future work. By using numerical analysis and ray tracing methods, the relational equation between the front temperature field and the light spot of the rear optical lens was derived, thereby enabling the image quality of the optical system resulting from variations in the front dome temperature field to be evaluated.

### 2.1. Numeric Simulation Method

The heat conduction differential equation must be resolved to calculate the aircraft temperature field. This is difficult to achieve through analytical methods, so numeric simulation was used. Common numeric simulation methods include the thermal network method [7,8] and the FEM. The thermal network method has poor precision [9], so the FEM was used to ensure the precision of the results.

First, a numerical solution was found using FEA software [10] (ANSYS [11]). During FEA, the thermal-structural element type solid226 with second-order accuracy was used. After importing the model into ANSYS, meshes were generated with a built-in meshing tool. To ensure the high precision of the analysis, the density of the meshes was increased until there was no change in the results that could be attributed to changes in density. For the front dome, the surface heat flux density showed a certain gradient. To accurately describe the gradient load, high-density meshes were generated on the front dome (element density > 5/cm^3^). The rear optical lens has a regular shape and a small temperature gradient, so the density was set at 0.4/cm^3^ to save calculation time. The aerodynamic heat flux density was applied to the model to conduct transient analysis, and the temperature field distribution was then derived after flying for different durations.

An enormous amount of time is needed to perform accurate FEA on the aerodynamic heat of an aircraft, because in an open flow field, a space that is thousands of times bigger than the aircraft should be included for mesh generation, which can cause memory overflow and consume an indefinite amount of time. During the design process, the system must be modified and evaluated iteratively, so simplifying the calculation process could profoundly reduce the design cycle. The most common approach is the so-called engineering method [1], which, although sacrificing a certain amount of precision, can save much calculation time and resources.

In the engineering method, the parameters with less influence on the results were simplified based on theoretical analysis, thereby producing empirical and semi-empirical equations based on the FEA experiment. Common engineering methods for aerodynamic heat calculation include the Lees equation [12], the Fay-Riddell equation [13] and the Scala equation [14]. These equations, which provide the physical and mathematical modelling for hypervelocity convective aerodynamic heating, can “avoid the need to deal with the chemistry of finite reaction rates” [15], wherein recombination would lag behind the changes instead of instantly responding to the rapidly varying flow conditions across the thickness of a boundary layer, and be tested with Mach numbers as high as 17.5. The Fay-Riddell equation is highly precise but very complicated. The Lees equation provides a comprehensive method of assessing both the stagnation and non-stagnation point regions. The Lees equation was later simplified, producing the modified Lees equation, as shown in Equation (1):

(1) $$q_{ws}=2.373\times 10^{-7}{\left(\frac{\gamma_{\infty }-1}{\gamma_{\infty }}\right)}^{0.25}{\left(\frac{\gamma +1}{\gamma -1}\right)}^{0.25}{\rho_{\infty }}^{0.5}{u_{\infty }}^{3}{R_{N}}^{-0.5}$$

Here, *q_ws_* is the heat flux density of the stagnation point (kW/m^2^), *R_N_* is the radius of curvature of the dome (m), *ρ*_∞_ is air density (kg/m^3^), *u*_∞_ is air speed (m/s), *γ*_∞_ is the specific heat ratio of inlet air flow (1.4) and *γ* is 1.4 for a perfect gas. At an attach angle of 0, the normal pressure of a cylindrical aircraft is 0, so there is no aerodynamic heat at the side surface. The modified Lees equation was used here because calculating the aerodynamic heat at non-stagnation points was necessary.

### 2.2. Parameter Setting for Ray Tracing in the Temperature Field

The influences of the temperature field on the physical geometry of the optical system are due to two main factors: (1) changes in lens thickness and lens surface profile; and (2) changes in air refractive index and the index of refraction of optical and mechanical structures. For target detection optical systems, an evaluation of optical system performance mainly concerns focusing performance, i.e., the light spot position and size received by the four-quadrant detector. If there is any defocusing due to parameter changes, positioning precision will be reduced, resulting in system failure.

The thermal load of an aircraft is primarily aerodynamic heat with an attach angle of 0, and if the distribution and aircraft structure are both rotational and symmetric, the internal temperature field has a radial gradient with an optical axis as the rotation centre. During thermal analysis of the airborne optical system, the gradient temperature field was introduced with the optical axis serving as the rotational axis. The temperature of the optical components at radius *R* can then be expressed as follows [16]:

(2) $$T\left(R\right)\approx T_{0}+d_{1}R^{2},$$

where *T*_0_ is the temperature at the optical axis and *d*_1_ is a coefficient determined by the linear least square method of sampling temperature. Thus, the fitted temperature distribution function can be obtained based on Equation (2). The refractive index *n*(*R*) at radial height *R* can be determined based on Equation (3):

(3) $$n\left(R\right)=n_{20}+\left(T\left(R\right)-20\right)\frac{dn}{dt}\approx n_{20}+\left(T_{0}+d_{1}R^{2}-20\right)\frac{dn}{dt}.$$

**Figure 2:**
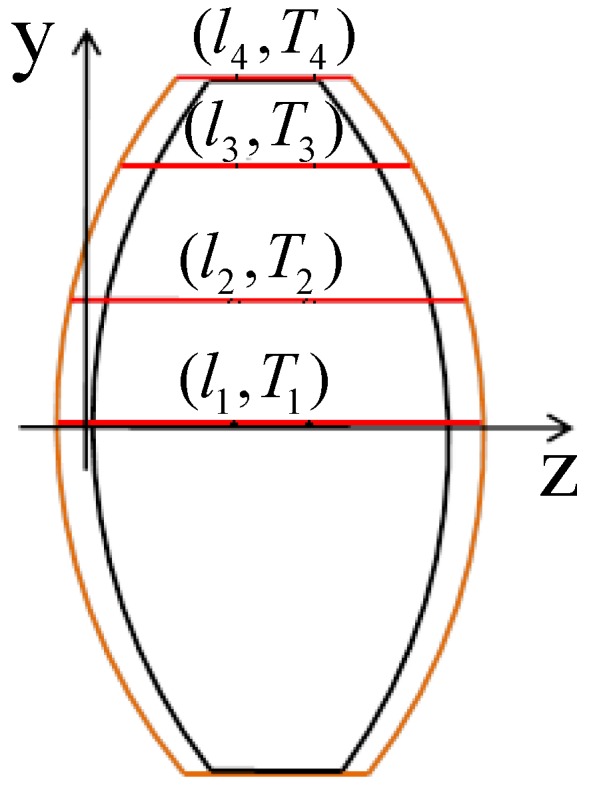
Radial gradient and profile of optical components.

The subscript 20 in *n*_20_ refers to the standard index of refraction obtained at 20 °C. The change in the surface profile can be calculated based on the simple *y-z* coordinate model, as shown in Figure 2. As given above, the airborne optical system of a supersonic aircraft with an attach angle of 0 has a radial internal temperature field, so the points on the component surface with the same *y* coordinate show equal levels of variation after changes in the thermal environment. The differences in the *z* coordinates can be calculated based on the thermal expansion:

(4) $$l=l_{0}(1+TCE\cdot \Delta T),$$

where *l*_0_ is the length of the optical component before thermal expansion and *l* is the length after expansion. *TCE* is the thermal expansion coefficient and Δ*T* the temperature change. Changes in dome geometry and performance can be obtained according to Equations (2)–(4).

**Figure 3:**
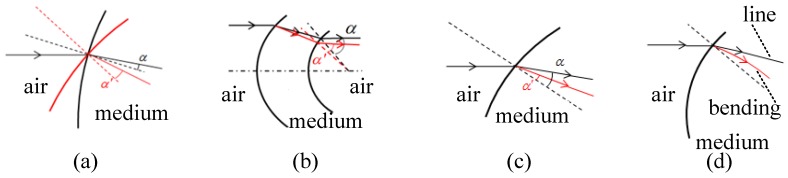
Temperature and ray propagation. (**a**) Thermally induced surface slope affects the emergent angle; (**b**) changing the emergent angle of the front surface affects that of the rear surface; (**c**) changing the refractive index at the incident point affects the emergent angle; (**d**) the radial gradient refractive index affects the propagation bending path.

Based on the above discussion and on theoretical deduction and physical simulation, the semi-empirical equation of change in the emergent ray angle Δ*α* was obtained for conditions before and after environmental change. Analysis of the changes in the front dome, in terms of physical geometry and refraction, demonstrated that influences on the direction of the emergent ray were mainly of four types, as shown in Figure 3: (a) the thermally induced surface slope changed the direction of normal lines and thus affected the emergent angle; (b) changing the emergent angle of the first surface affected the incident angle of the second surface; (c) changing the refractive index at the incident and emergent points of two surfaces affected the deflection angle; and (d) the radial gradient of the refractive index caused the propagation path to bend. According to the optical sensitivity method [6], the total change of ray propagation direction was equal to the sum of the four changes above. The deduction of the total angle change of emergent ray Δα is described in Appendix B.

**Figure 4:**
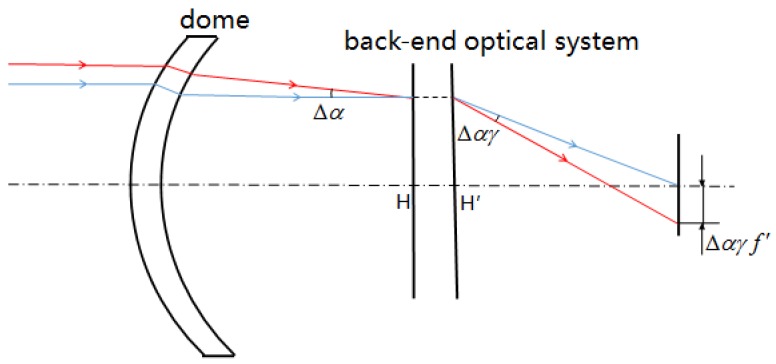
Schematic diagram of the equivalent optical system of an airborne optical system.

Based on the equation of the dome emergent ray angle change ((B11); see Appendix B), the change of light spot radius can be further obtained. It was assumed that the change in the emergent ray at the edge of the dome aperture was Δ*α* , the focus of the rear optical lens was *f*’, and the angular magnification was γ, as shown in Figure 4. The incident light ray of the main plane of the object changed by Δ*α* , and the emergent light ray of the image main plane changed by Δαγ, so the image spot position change caused by this angle change was Δ*αγf’*. When there was a large change in the temperature field, the angle change of the emergent light ray at the aperture edge was significantly greater than that at the centre, which caused the light ray at the aperture edge to replace that at 0.707 of the aperture and form a marginal image spot. During optical system design, a spherical aberration correction of the marginal ray is commonly made, so the image spot displacement of the marginal ray is equal to its coordinates. Hence, the light spot radius after thermal environment change can be expressed as follows:

(5) r=Δαγf′.

**Figure 5:**
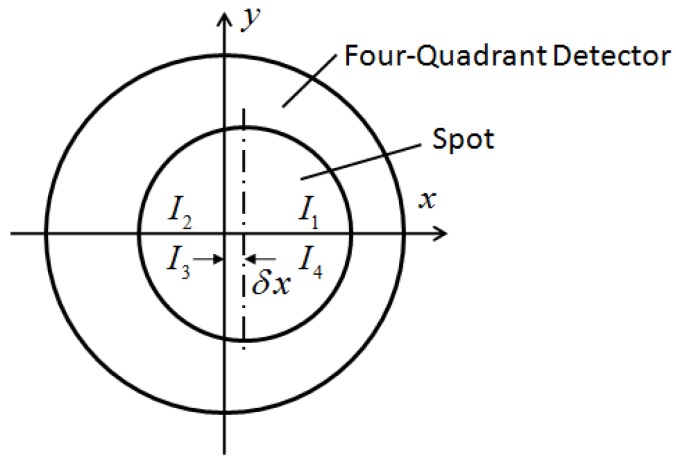
Structure of the four-quadrant detector.

As stated above, changes in the radius of the light spot can affect the solution precision of the four-quadrant detector. The structure and focal spot of the four-quadrant detector are illustrated in Figure 5. The photosensitive surface transforms light into current. During the calculation, current was used to replace light. The centre of mass method was used to resolve the position. Here, when the centre of the light spot approaches the centre of the detector, the light intensity of every quadrant is proportional to the spot area of every quadrant:

(6) $$\begin{array}{c} \Delta x=k\frac{\left(I_{1}+I_{4}\right)-\left(I_{2}+I_{3}\right)}{I_{1}+I_{2}+I_{3}+I_{4}}\\ \Delta y=k\frac{\left(I_{1}+I_{2}\right)-\left(I_{3}+I_{4}\right)}{I_{1}+I_{2}+I_{3}+I_{4}} \end{array},$$

where Δ*x* and Δ*y* are the coordinates of the spot centre. *I_i_* is the light intensity of each quadrant and *k* is a scale factor. Assuming that *S_i_* is the spot area of each quadrant, *δx* and *δy* are the coordinates of the spot centre and *r* is the radius of the spot, then the following is true:

(7) $$\begin{array}{c} \Delta x=k\frac{\left(S_{1}+S_{4}\right)-\left(S_{2}+S_{3}\right)}{S_{1}+S_{2}+S_{3}+S_{4}}=k\frac{4r\delta x}{\pi r^{2}}=\frac{4k\delta x}{\pi r}\\ \Delta y=k\frac{\left(S_{1}+S_{2}\right)-\left(S_{3}+S_{4}\right)}{S_{1}+S_{2}+S_{3}+S_{4}}=k\frac{4r\delta y}{\pi r^{2}}=\frac{4k\delta y}{\pi r} \end{array},$$

where *k* is related to the parameters of the detector and the optical system. After system design and model selection, the proper *k* value can be determined via experimentation, so the calculation value equals the actual value. According to Equation (7), when the spot area changes the radius *r* also changes. The precision in target positioning is positively proportional to the change in the reciprocal spot radius *r,* so the positioning and calculation precision will be affected. The requirement of the spot radius can be derived from the requirements for the precision of the four-quadrant detector.

## 3. Results

In this section, the empirical equations in the temperature field (which includes airborne optical system structure, flight speed and flight time under thermal diffusion conditions), are investigated based on numeric simulations using the flying and structural parameters of the optical systems used for navigation guidance and control in supersonic aircraft. As stated above, during the integrated analysis of the optical system, the parameters of the front dome and the rear optical lens are both taken into account to ensure that the rear lens is not influenced. Changes in the performance of the rear lens can lead to further decreases in the performance of the overall system. The influence of structural parameters on thermal diffusion distance were therefore investigated based on FEA and factor experiments to derive the empirical equations for analysing the effects of thermal diffusion on the airborne optical system.

The thermal load of optical systems for navigation guidance and control in supersonic aircraft is concentrated on the front dome, which directly exchanges heat with the outside. The heat of the rear optical lens is conducted via the aircraft from the dome, so unlike the dome, it does not always suffer from large effects under all conditions. Only when the change in temperature reaches a certain magnitude will the performance of the system be significantly affected. Therefore, when investigating the influence of different factors on the aircraft’s internal temperature field, the effects on the rear optical lens should be evaluated early on in the design. The dome parameters and rear structural parameters can then be optimised, based on the analysis of the relationship between the position of the isothermal surface at a specific temperature and the flying and structural parameters. An athermal design under thermal diffusion conditions is then enabled and the systematic optimisation of structural parameters for the dome and optical lens can prevent thermal diffusion from the dome to the rear optical lens.

FEA and factor experiments were carried out for aircraft structural and flying parameters based on numeric simulation to derive empirical equations involving airborne optical system structure, flight speed and flight time under thermal diffusion conditions. First, to explore the influence of dome curvature, aircraft speed and time on the performance of the optical system, three simulation experiments were conducted in ANSYS, illustrating the aircraft temperature field distribution under different conditions. Using a constant flight speed (400 m/s), material thermal conductivity (800 W*/*m·K), specific heat (900 J/kg·K) and material density (2000 kg/m^3^), the dome radius of curvature and flight time were adjusted to assess the rule of thermal diffusion inside the aircraft. Four groups of data were tested and the results are given in Table 1. The thermal diffusion distance is the distance between the isothermal surface and the front of the cylindrical shell when the temperature increases by 2 °C. Although a variation of the dome radius of curvature was found to influence aerodynamic heat flux, the rear of the aircraft would not be affected over a flight time of under 50 s, as shown in Table 1. Longer flight times led to significant increases in the thermal diffusion to the rear end.

Flight speed influences aerodynamic heat, so an analysis of flight speed was achieved by changing the aerodynamic thermal load. Similarly, four groups with different dome radius of curvature and flight time were selected. For each group, FEA was performed at three different flight speeds and the corresponding temperature field distribution was derived. Table 2 shows the temperature increase at the stagnation points. The modified Lees equation indicated a proportional relationship between aerodynamic heat flux and cubic flight speed. Table 2 shows that when only flight speed (aerodynamic thermal load) was changed, the increase in temperature at the stagnation points was proportional to the aerodynamic heat, which also applies to all other points of the aircraft because the thermal conduction differential equation under conditions of no internal thermal source is a linear differential equation with constant coefficients, so the whole system becomes linear:

(8) $$\frac{\partial T}{\partial t}=\frac{k}{c\rho }\left(\frac{\partial^{2}T}{\partial x^{2}}+\frac{\partial^{2}T}{\partial y^{2}}+\frac{\partial^{2}T}{\partial z^{2}}\right),$$

where *T* is temperature, *k* is thermal conductivity factor, *t* is time, *c* is specific heat, *ρ* is density and (*x,y,z*) are Cartesian coordinates.

As shown in Table 2, for a specific aircraft flying for the same amount of time, if the rear optical lens is not affected at low flight speeds (i.e., there are no changes in temperature at the rear end), changes in temperature at high speed will remain small. However, if the system is affected at low speed, the temperature change will be more significant at high flight speeds.

**Figure 6:**
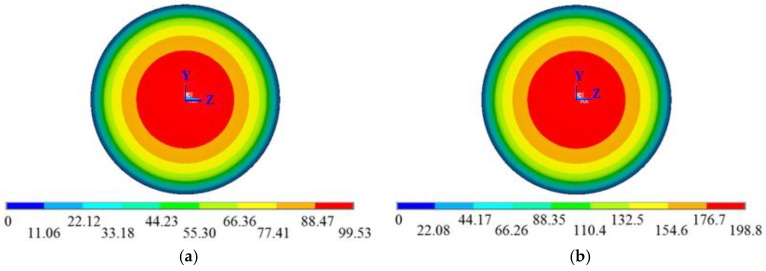
Temperature field distribution at different *cρ*: (**a**) 1800; (**b**) 900.

The material properties of the aircraft (i.e., specific heat, density and thermal conductivity factors) also influenced the degree of thermal diffusion. Heat is expressed as follows:

(9) Q=cmΔT,

where *Q* is the heat inflow of the material, *c* is specific heat, m is the mass and Δ*T* is the change in temperature. The mass *m* is proportional to density *ρ* at a fixed volume, so the change in temperature is proportional to the change in heat when the value of the product *cρ* is constant. Combining Equations (8) and (9), the product of specific heat and material density *cρ* can be considered as a single parameter in the analysis. When *cρ* increased, the speed of thermal diffusion decreased and the position of the isothermal surface was constant and close to the dome, as shown in Figure 6. The thermal diffusion otherwise accelerated as *cρ* decreased. The following FEA experiment was carried out to verify the speculation. The flight time, speed, dome radius and thermal conductivity factor were set to 20 s, 400 m/s, 530 mm and 800 W*/*m·K, respectively. Three sets of different specific heat and density were used during the simulation. To illustrate that the two parameters could be combined, their values were changed during every simulation. In the first group, the specific heat was 600 J/kg∙K and the density was 6000 kg/m^3^; in the second, the specific heat was 450 J/kg∙K and the density was 4000 kg/m^3^; and in the third, the specific heat was 900 J/kg∙K and the density was 1000 kg/m^3^. The increases in temperature at the stagnation points are listed in Table 3. The results indicate that the aircraft temperatures at different positions were inversely related to *cρ*. The influence of 1/*cρ* on the temperature field was observed to be similar to that of aerodynamic heat. Therefore, the change in cρ can be considered to be approximately equivalent to the change in aerodynamic heat; when 1/*cρ* was changed by a certain ratio, the aerodynamic heat was considered to change by the same ratio.

Equation (8) describes the influence of the thermal conductivity factor *k* on the thermal diffusion distance. With increasing *k*, the rate of change of temperature increases accordingly, resulting in fast thermal diffusion to the rear end.

In contrast, decreases in the thermal conductivity factor can cause a concentration of heat at the front, resulting in low temperatures near the rear end, as confirmed by the FEA experiment on the influence of thermal conductivity factor on the temperature field. Different thermal conductivity factors were investigated at constant values of flight time (20 s), flight speed (400 m/s), dome radius (530 mm), specific heat (900 J/kg∙K) and density (2000 kg/m^3^). The results are shown in Table 4.

Based on the FEA results of the simulation, the least square method was used to determine the empirical Equation (10), or the relational equation between thermal diffusion and factors such as dome radius, flight time and material thermal conductivity. As shown in Table 2, if the rear lens is not affected at low speeds, it is not affected at high speeds. Therefore the flight speed and equivalent specific heat and density were not included in the formula. However, the temperature at the thermal diffusion distance can be estimated based on flight speed, material density and specific heat by using the FEA experiments and the function fitting method.

(10) $$\begin{array}{l} F(r,t,k)=10\times [55.755\sqrt{t}+1.507t-112.380+(0.7645\sqrt{k}-8.2896e-4k-20.883)\\ \times (3.005\sqrt{t}+0.0414t-4.233)]+\left(0.02903+0.001016t-0.0184\sqrt{t}\right)\left(r-530\right) \end{array},$$

where *F*(*r,t,k*) is the thermal diffusion distance, *r* is dome outer radius (mm), *t* is flight time (s) and *k* is the material’s thermal conductivity factor (W/m∙K).

The same method was used to investigate the temperature field of the front dome, producing the empirical Equation (11) of the temperature gradient:

(11) $$\begin{array}{l} T(r,t,k,c,\rho ,v)=\\ \left[\left(0.7602t+42.7168\sqrt{t}-59.7262\sqrt[{3}]{t}\right)-\left(0.2313t-0.6042\sqrt{t}-0.5830\sqrt[{3}]{t}\right)\frac{k}{100}\right]\\ \;\times \frac{1.8e6}{c\rho }{\left(\frac{v}{400}\right)}^{3}{\left(\frac{r}{530}\right)}^{-0.8137-9.3e-4t} \end{array},$$

where *T* is the temperature gradient value (K) at 0 and 250 mm of the dome radius; the definitions of *r*, *t* and *k* are as above. *v* is speed (m/s), *c* is specific heat (J/kg∙K) and *ρ* is density (kg/m^3^). The results indicated that all flying parameters and system structural parameters significantly influence the temperature field.

## 4. Discussion

To validate the above analysis, a specific system layout was used to study the applicability of the integrated thermal-mechanical-thermal analysis method. The flying parameters and aircraft structural parameters were as follows: flight time 35 s, flight speed 500 m/s, front surface radius of the dome 670 mm, material density 3600 kg/m^3^, thermal conductivity factor 400 W*/*m·K, specific heat 700 J/kg∙K, angle magnification of rear optical lens 1.25 and focal length 17.9 mm.

The empirical equations in Section 3 were then used to calculate the temperature gradient of the front dome and to determine whether the rear system was affected. The equations in Section 2 and Appendix B were used to calculate the influence of the temperature field on the light spot radius. According to Equation (10), the rear thermal diffusion distance was 281.3 mm and the corresponding temperature was 2.79 °C, so it can be concluded that the rear lens was not affected. The system performance can thus be determined from the front temperature gradient and the original rear lens structure. However, due to the large dome surface temperature gradient of 43.1 °C in the front, the performance of the whole optical system was greatly affected. Based on Equation (5) and the equations in Appendix B, the light spot diameter was found to be 0.0781 mm. The duration of the calculation can be expected to be less than 1 s.

For method validation and comparison, experiments using ANSYS software were also carried out for environmental analysis. The element density of the dome was 5.08 elements/cm^3^, and that of the cylindrical shell was 0.5 element/cm^3^. A computer with a dual-core i3-3220 processor, 3.3 GHz CPU and 4 GB memory was used and required a computation time of 7 h. Code V was then used for performance analysis [16] according to the temperature field distribution parameters obtained in ANSYS. After setting the thermal refractive index coefficient, the surface profile and refractive index at every position were calculated using CODEV software.

**Figure 7:**
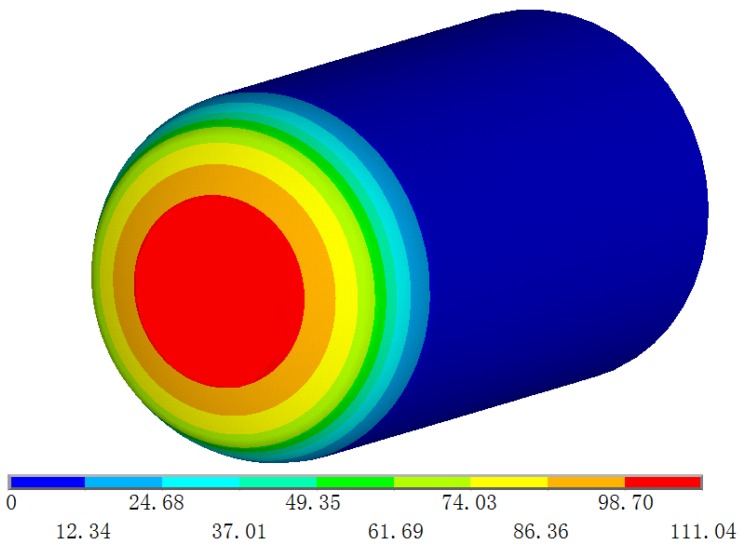
Aircraft temperature field.

**Figure 8:**
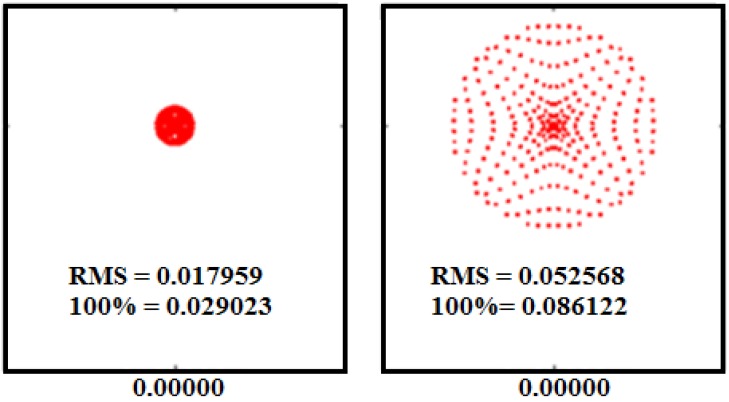
Spot diagram of optical system before and after receiving heat.

The aircraft temperature field is shown in Figure 7. The FEA experiment results indicated that the dome temperature gradient was 44.15 °C and that the 2.79 °C isothermal surface of the rear system was 282.9 mm from the front of the cylindrical aircraft, and did not diffuse to the rear system. The large dome temperature gradient of 43.1 °C also affected the system performance. The tangent value of the angle formed by the emergent ray from the dome back surface that passes the incident ray aperture margin and the optical axis decreased from 0.00554 to 0.00367, and the spot diameter increased from 0.029 mm to 0.086 mm at 0° field of view, as shown in Figure 8.

The experiment above proves the effectiveness of the new method. Based on the light spot size obtained from FEA and image quality analysis, the time required for the calculation decreased from 7 h to less than 1 s. Using the empirical equations, the relative error in light spot size was 9.2%. This method was thus found to be beneficial for rapid validation during the early stages of the design process. When approaching the target, the method could be replaced with the traditional method to ensure precise validation.

## 5. Conclusions

During the flight of supersonic aircraft, the aerodynamic heat generated causes a large temperature gradient on the dome, which brings the optical system out of focus and thus greatly affects performance. Athermal design and validation is therefore necessary. In this study, the influence of the dome temperature field on the light ray emergent angle was investigated, based on relational equations of the influence of the temperature field on the geometry and performance of the optical components. The influence of the front dome temperature field on the system light spot and positioning precision was derived. A method combining FEA and factor analysis was proposed to obtain the empirical equations between the front temperature gradient and rear thermal diffusion of the airborne optical system and the system structure, flight speed and time. An empirical equation suitable for assessing the influences on the rear optical lens was then developed, as was an equation to calculate the front temperature gradient. The results showed that although flight speed and the dome radius of curvature affected the value of aerodynamic heat, their influences on the temperature field of the rear optical lens were not significant in the short term. However, flight time and the thermal conductivity factor had significant effects on the degree of thermal diffusion. For the front system, no flying parameter or structural parameter was negligible. The empirical equations enabled rapid evaluation of the influence of the thermal environment on the performance of the system. The experiment indicated that the rapid algorithm could dramatically reduce the calculation time from 7 h to less than 1 s while providing a measure of the relative error in light spot size (for example, 9.2%). In this study, it was assumed that the system worked with an attach angle of 0; other conditions (for example, with a variable attach angle) of the flow around the modelled sensor will be the subject of future work.

## Figures and Tables

**Table 1:** Thermal diffusion at varying dome radiuses and flight times ^1^.

	Flight Time	20	30	40	50
Dome Radius					
460		169.1	241.6	305.0	362.5
530		166.4	239.1	300.7	357.2
600		164.2	234.7	297.7	353.1
880		154.9	224.6	284.5	339.6

^1^ Units: dome radius: mm, flight time: s, thermal diffusion: mm.

**Table 2:** Flight speed and stagnation temperature ^1^.

	Dome Radius/Flight Time	460/20	530/20	600/30	880/40
Flight Speed					
400		113.8	99.5	129.3	127.1
503.7		227.3	198.8	258.3	253.7
576.6		340.9	298.2	387.4	380.6

^1^ Units: flight speed: m/s, dome radius: mm, flight time: s, increase of stagnation temperature: °C.

**Table 3:** Effect of material density and specific heat on stagnation temperature ^1^.

cρ	3600	1800	900
Increase in stagnation temperature	49.8	99.5	198.8

^1^ Units: $c\rho :\;\mathrm{kJ}/\left(K\cdot m^{3}\right)$, increase of stagnation temperature: K.

**Table 4:** Material thermal conductivity and aircraft temperature field ^1^.

Thermal Conductivity	100	400	800	1600
Increase in stagnation temperature	124.91	106.07	99.53	88.75
Thermal diffusion distance	32.8	108.4	166.4	250.6

^1^ Units: thermal conductivity: W/m K, increase of stagnation temperature: K, thermal diffusion distance: mm.

**Figure A1:**
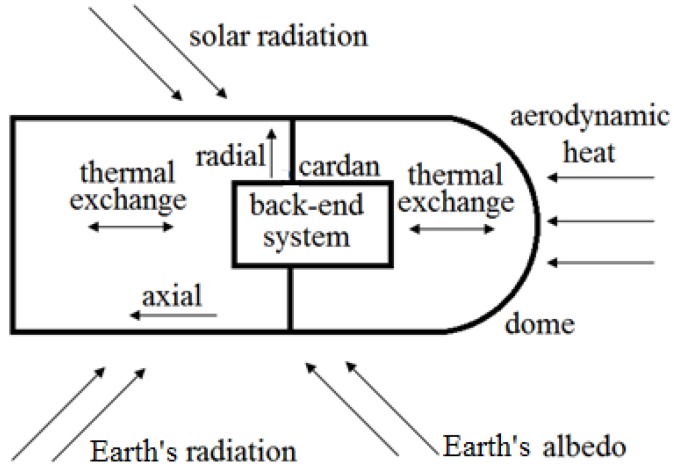
The thermal environment for an airborne optical system.

**Figure B1:**
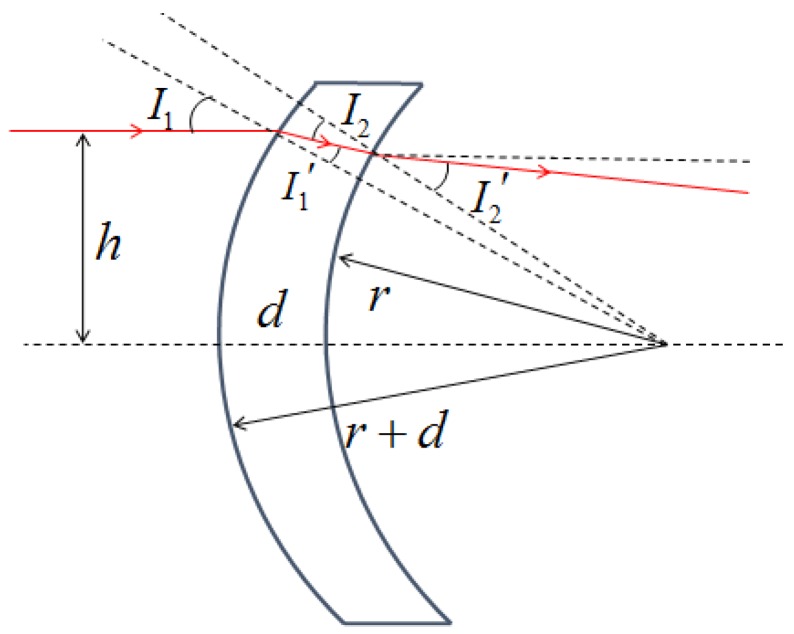
Light ray tracing model of the dome.

## Appendix A

The temperature fields of supersonic aircraft in flight are affected by aspects of the complex thermal environment: aerodynamic heating, solar radiation, Earth’s albedo, Earth’s radiation and thermal exchange. (a) Aerodynamic heat consists of two parts, the most important for high-speed aircraft being violently compressed air producing extreme heat, and the other being friction heat; (b) Solar radiation. The sun has a surface temperature of about 6000 K. The radiation power density was calculated according to the Stepan-Boltzmann equation (Equation (A1) below), in which $E_{b}$ is the solar radiation constant, σ is the Stepan-Boltzmann constant and T is the absolute temperature on the sun’s surface. By using Equation (A1) and the Earth–sun distance, the solar constant *S* is set at 1353 W/m^2^. Considering the effect of atmospheric absorption, which is 20%, and the aircraft surface-reflected radiation as the flying aircraft is mainly under the slanting radiation condition of the sun, the power density of the received radiation is less than 600 W/m^2^; (c) Earth’s albedo. The Earth receives part of the solar radiation reflection. The average albedo constant α is 0.35. The power density of the Earth’s albedo $E_{r}$ is calculated with Equation (A2); (d) Earth’s radiation. Calculating heat radiation from the Earth is complex. The Earth is set as a 250 K black body, and its total radiation is obtained as 221 W/m^2^ by using Equation (A1); (e) Thermal exchange. In addition to the dome surface radiation and the heat exchange from other surfaces, air convection can also cause thermal exchange. The thermal flux is calculated with Equation (A3), where h is the convection coefficient, generally $5–25\;W/\left(m^{2}\cdot K\right)$ in air. ΔT is the temperature gradient at the solid–air interface.

(A1) $$E_{b}=\sigma T^{4}$$

(A2) $$E_{r}=\alpha E_{b}$$

(A3) q=hΔT

Figure A1 shows that the aerodynamic heat mainly focuses on the dome surface. The power density of the aerodynamic heat is 22.4–1192 kW/m^2^ at the stagnation point when the flight speed is 1–5 Ma and the dome radius is 0.055–1.0 m. The solar radiation focuses on the top surface. The Earth’s albedo and radiation focus on the bottom surface. The power densities of these radiations are no more than 200 W/m^2^, and the power density of the inner thermal exchange is much lower. Thus aerodynamic heat is the major source of heat load for the optical systems for navigation guidance and control in supersonic aircraft.

## Appendix B

This appendix provides a deduction of the exit ray angle change of the rear dome surface, as illustrated in Figure B1. Hypothetically, the incident angle and emergent angle of the first surface are $I_{1}$ and $I_{1}^{\prime }$, respectively, and of the second surface, $I_{2}$ and $I_{2}^{\prime }$, respectively. The index of refraction of air is *n*, and of the material *n*^′^. The change in temperature at the incident positions of the first and second surfaces are Δ*T*_1_ and Δ*T*_2_, respectively. The height of incidence is *h*, the first surface thickness is *d*, and the radius of curvature of the second surface is *r*. The two dome surfaces are usually eccentric circles, so the radius of curvature of the second surface is *r* + *d*, in general, *d* ≪ *r*.

The equation for light ray emergent angle without environmental change was first deducted.

The sine value of the incident angle of the first surface is as follows:

(B1) $$\sin I_{1}=\frac{h}{r+d}.$$

The sine value of the emergent angle of the first surface is as follows:

(B2) $$\sin I_{1}^{\prime }=\frac{h}{r+d}\frac{n}{n\prime }.$$

The sine value of the incident angle of the second surface is as follows:

(B3) $$\sin I_{2}=\frac{h}{r}\frac{n}{n\prime }.$$

The sine value of the emergent angle of the second surface is as follows:

(B4) $$\sin I_{2}^{\prime }=\frac{h}{r}.$$

Hence, the light ray emergent angle is as follows:

(B5) $$U=I_{1}+I_{2}-I_{1}^{\prime }-I_{2}^{\prime }.$$

From Taylor expansion of inverse trigonometric functions, the following can be found:

(B6) $$U=\left(\frac{n}{n^{\prime }}-1\right)\left[\frac{hd}{r\left(r+d\right)}+\frac{1}{2}\frac{h^{3}d}{r^{2}{\left(r+d\right)}^{2}}\right].$$

Changes in temperature can cause changes in the geometry of the surfaces of optical components and index of refraction, both of which may change the light ray emergent angle. Their individual influence on light rays was deducted separately. The thermal refractive index coefficient is written as *dn*/*dt*, and thermal expansion coefficient is written as *TCE*.

1. Index of refraction on light ray

The change in the sine value of emergent angle of the first surface:

(B7) $$\Delta \sin I_{1}^{\prime }=\frac{h}{r+d}\left(\frac{n}{n\prime +\frac{dn}{dt}\Delta T_{1}}-\frac{n}{n\prime }\right).$$

Hence, the change of emergent angle is as follows:

(B8) $$\Delta I_{1}^{\prime }=\frac{\Delta \sin I_{1}^{\prime }}{-\cos I_{1}^{\prime }}.$$

The change in the incident angle of the second surface is in three parts: (1) changes from emergent angle variation of the first surface; (2) deflection of light rays during propagation from the first surface to the second surface, due to the gradient index of refraction of component surfaces; (3) change in component surface normal lines due to incident point change. The change in the angle during the second part is written as Δ*θ*.

(B9) $$\Delta \theta =\left(\frac{n^{\prime }+\frac{dn}{dt}\Delta T_{1}}{n^{\prime }+\frac{dn}{dt}\Delta T_{2}}-1\right)\left(\frac{\pi }{2}-\arcsin(\frac{h}{r+d})+I_{1}^{\prime }\right).$$

Then the total change of incident angle of the second surface is as follows:

(B10) $$\Delta I_{2}=\left(\Delta I_{1}^{\prime }+\Delta \theta \right)\left(1+\frac{d}{r}\right).$$

Hence, the change of emergent value of the second surface (i.e., change of emergent light ray) is

(B11) $$\Delta U=\arcsin\left[\sin\left(\arcsin\frac{hn}{rn^{\prime }}-\Delta I_{2}\right)\frac{n^{\prime }+\frac{dn}{dt}\Delta T_{2}}{n}\right]-\arcsin\frac{h}{r}.$$

According to thermo-optical sensitivity theory and the practical physical simulation experiment, the changes in emergent light ray are proportional to the temperature gradient between the component’s centre and margin. Replacing Δ*T*_1_ and Δ*T*_2_ in (xi) with the function of temperature gradient does not affect the results. The following replacement was performed to render the equation suitable for conditions with different apertures.

Assuming that the temperature gradient between the component centre and a specific value at the radius of *R* (for example, 250 mm) is ∇*T*, then Δ*T*_1_ = (*h*^2^/*R*^2^) ∇*T*. The calculation of Δ*T*_2_ requires the height of incidence of the second surface, *h*_2_. As emergent light is generally close to the normal lines and the dome thickness *d* is small, *d* can be used to replace the axial propagation distance within the dome. So *h*_2_ = *h* − *d*sin$\left(\arcsin\frac{h}{r+d}-\arcsin I_{1}^{\prime }\right)$, and ΔT2=h22R2∇T. Herein, when the dome structure is determined, the function between light emergent angle and temperature gradient can be obtained.

2. Surface geometry and light rays

Changes in surface geometry cause variations in the slope of the surface profile. These changes were calculated in this study using a numerical differentiation algorithm. Taking the first surface, for example, $z_{h+\Delta h}^{1}$ is the arc height of the position on the first surface that is vertically *h* + Δ*h* from the optical axis. Similarly, $z_{h+\Delta h}^{1}$ is *h* − Δ*h* from the optical axis. Then, the slope before the temperature change is as follows:

(B12) $$K_{10}=\underset{\Delta h\to 0}{\lim}\frac{2\Delta h}{z_{h+\Delta h}^{1}-z_{h-\Delta h}^{1}}=\frac{\sqrt{r^{2}-h^{2}}}{h}.$$

The change in slope was calculated based on the thermal expansion model in Section 2. First, the interval between *h* + Δ*h* and *h* − Δ*h* was calculated and recorded as follows:

(B13) $$d_{h+\Delta h}=\sqrt{r^{2}-{\left(h+\Delta h\right)}^{2}}-\sqrt{{\left(r-d\right)}^{2}-{\left(h+\Delta h\right)}^{2}},$$

(B14) $$d_{h-\Delta h}=\sqrt{r^{2}-{\left(h-\Delta h\right)}^{2}}-\sqrt{{\left(r-d\right)}^{2}-{\left(h-\Delta h\right)}^{2}}.$$

Then, the slope before temperature change was as follows:

(B15) $$K_{11}=\underset{\Delta h\to 0}{\lim}\frac{2\Delta h\left(1+TCE\Delta T_{1}\right)}{z_{h+\Delta h}^{1}-z_{h-\Delta h}^{1}-\frac{1}{2}TCE\Delta T\left(d_{h+\Delta h}-d_{h-\Delta h}\right)}.$$

The slope change is Δ*K* = *K*_11_ − *K*_10_. By substituting Equations (B13)–(B15) and considering *TCE* ≪ *d* ≪ *r*, the following can be determined:

(B16) $$\Delta K=\frac{\sqrt{r^{2}-h^{2}}}{h}TCE\Delta T.$$

The change of incident angle because of the slope change was as follows:

(B17) $$\Delta I_{1}=\cos^{2}I_{1}\cdot \Delta K.$$

The changes in normal lines and aperture angle (the angle between the emergent line and the optical axis) of emergent light of the first surface due to the slope change were as follows:

(B18) $$\Delta U_{1}=\Delta I_{1}+\Delta I_{1}^{\prime }=\Delta I_{1}\left(1+\frac{n}{n^{\prime }}\cos I_{1}\right).$$

The change in the emergent light ray finally consisted of two parts: (1) change in the emergent angle due to the slope change of the second surface; (2) change in the emergent angle due to the varying incident angle caused by changes in the aperture angle of the emergent light ray of the first surface. The first part was similar to the change in aperture angle of emergent light rays on the first surface:

(B19) $$\Delta U_{21}=\Delta I_{2}+\Delta I_{2}^{\prime }=\Delta I_{1}\left(1+\frac{n^{\prime }}{n}\cos I_{2}\right).$$

The second part was:

(B20) $$\Delta U_{22}=\Delta I_{2}\frac{n^{\prime }\cos I_{2}}{n\left(1-\sqrt{\frac{{n^{\prime }}^{2}\sin^{2}I_{2}}{n^{2}}}\right)},$$

where Δ*I*_2_ is the incident angle of the second surface:

(B21) $$\Delta I_{2}=\Delta U_{1}(1+\frac{d}{r}).$$

Hence, the total change of emergent light ray due to surface profile change was as follows:

(B22) $$\Delta U_{2}=\Delta U_{21}+\Delta U_{22}.$$
